# A hyper-acute immune hemolytic anemia induced by contrast medium was successfully treated with eculizumab: a case report

**DOI:** 10.3389/fimmu.2025.1464014

**Published:** 2025-02-11

**Authors:** Sabine Hermann, Karina Althaus, Migdat Mustafi, Beate Mayer, Peter Rosenberger, Helene Anna Haeberle, Alice Bernard

**Affiliations:** ^1^ Department of Anaestesiology and Intensive Care Medicine, University Hospital Tuebingen, Tuebingen, Germany; ^2^ Clinical and Experimental Transfusion Medicine, University Hospital Tuebingen, Tuebingen, Germany; ^3^ Department of Thoracic and Vascular Surgery, University Hospital Saarland, Saarbruecken, Germany; ^4^ Insitutition of Transfusion Medicine, Charité, Berlin, Germany

**Keywords:** drug-induced hemolysis, contrast medium-induced hemolysis, eculizumab, hemolytic anemia, immunological hemolysis

## Abstract

Contrast medium is frequently associated with allergic reactions and kidney dysfunction. However, contrast media can induce hemolytic anemia with a broad spectrum of hemolytic manifestations. We report a 38-year-old patient with very severe immune hemolytic anemia after the application of iomeprol due to a CT of the thorax/abdomen. In this case report, we illustrate the diagnostics and treatment of life-threatening hemolytic anemia induced by a contrast medium that was successfully treated with eculizumab.

## 1 Introduction

Contrast medium (CM) can cause drug-induced hemolytic anemia with a broad spectrum of hemolytic manifestation; in cases of minimal hemolysis, it is probably often overlooked and undiagnosed. More than 130 drugs (1) are known to possibly cause hemolytic anemia, among them the contrast media iohexol and iomeprol (1, 2). The hemolysis shows a varying degree of severity, and so the four known case reports (1–4) differ in clinical outcome, varying between hemodynamic stabilization after blood transfusion and death of the patient. Our patient experienced said hemolytic anemia to a life-threatening degree and could successfully be treated with eculizumab.

## 2 Case description

On the day of the surgery, a 38-year-old male patient received surgical mechanical aortic valve replacement and a Dacron interponate in the ascending aorta due to congenital bicuspid aortic valve and aortic isthmus stenosis. Detailed patient history is shown in **Supplementary Table S1**.

On the same day, a hemothorax with mediastinal shift appeared. A second thoracotomy accompanied by massive transfusion was necessary, and veno-arterial extracorporeal membrane oxygenation (VA-ECMO) had to be implanted because of a concomitant cardiogenic shock. Due to a massive Harlequin syndrome, cannulization was changed to veno-arterial venous extracorporeal membrane oxygenation (VAV-ECMO). After recovery of the cardiac function, the VA-ECMO could be removed 8 days later. Further on, two more bleeding episodes occurred, which had to be treated via video-assisted thoracoscopy and further blood transfusions.

On post-op day (POD) 21, fever (up 39.1°C), leukocytosis, and slightly elevated lactate were recognized, despite broad-spectrum anti-infective therapy with fluconazole, cefepime, and daptomycin. Meropenem was added—replacing cefepime—to extend the anti-infective therapy. Additionally, blood culture samples, urine samples, and tracheal secretion were taken for microbiological testing, and testing for viral infections [severe acute respiratory syndrome coronavirus 2 (SARS-CoV-2), influenza virus A, influenza virus B, cytomegalovirus (CMV), herpes simplex virus type 1 (HSV)-1, and HSV-2] was performed on POD 21 and POD 22. All results came back negative.

We performed a CT scan to rule out a septic focus.

After the CT scan, blood gas analysis showed a dramatic drop in hemoglobin concentration and lactic acidosis. Focused Assessement with Sonography for Trauma (FAST) sonography, transthoracic echocardiography, and chest X-ray revealed no result explaining these findings. Laboratory findings and the blood gas analysis before and after the CT scan are shown in **Table 1**. A second CT scan was performed to rule out active bleeding. Neither the first nor second CT scan exposed an explanation for the hemoglobin decrease or the lactic acidosis. Eventually, laboratory findings showed severe intravascular hemolysis (**Supplementary Figure S1A**). Skin efflorescence in terms of diffuse redness all over the body appeared 1 to 2 hours later due to sediments of hemoglobin and its metabolic product depositing into the skin.

**Table 1:** Laboratory findings and blood gas analysis before and after hemolysis.

Laboratory findings	Normal range	Before CT scan (POD 22)	After CT scan (POD 22)
Hemoglobin 1 g/dL	12.0–16.0	8.1	5.2
Hematocrit %	42.0–52.0	25.2	15.2
Erythrocytes Mio/µL	4.2–6.2	2.6	1.36
Platelet count 1,000/µL	150–450	318	265
Leukocyte 1/µL	4,100–11,800	17,960	43,520
aPTT sec	<40	64	41
INR		1.2	1.9
D-dimer µL/mL FE	0–0.5		>80
Creatinine mg/dL	0.6–1.1	1.5	2.2
Total bilirubin mg/dL	<1.1	1.9	6.5
CRP mg/dL	<0.5		8.27
PCT ng/mL	<0.1		32.849
IL-6 ng/L	0–4	9.2	11,458.4
CK U/L	<170	94	*)
LDH U/L	<250	769	*)
Free hemoglobin mg/dL	<10		455.19
Haptoglobin mg/dL	<6		<6
BGA (art.)			
FiO_2%_		30	30
Hb g/dL	12–18	8.4	5
pO_2_ mmHg	65–95	92.7	16
sO_2%_	>96	98	120
pH	7.3–7.45	7.47	7.42
pCO_2_ mmHg	35–45	48.3	46.7
HCO_3_^−^ mmol/L	21–26	33.4	29
Base excess (BE) mmol/L	−2.0 to 2.0	9.8	5.1
Lactate mmol/L	<1.8	2.0	7.2
Glucose mg/dL	70–100	166	224
Na^+^ mmol/L	135–145	143	141
K^+^ mmol/L	3.6–4.8	4.7	5.5
Cl^−^ mmol/L	95–105	102	101
AaDO_2_ mmHg	5–30	60.8	35.5
PaO_2_/FiO_2_		309	400

POD, post-op day; aPTT, activated partial thromboplastin time; INR, international normalized ratio; CRP, C-reactive protein; PCT, procalcitonin; CK, creatine kinase; LDH, lactate dehydrogenase; BGA, blood gas analysis.

^*)^ No result due to hemolytic plasma (as presented in **Supplementary Figure S1**).

Symptomatic treatment was initiated by hourly blood transfusion and substitution of coagulation factors according to laboratory findings.

Probable reasons and their likeliness were discussed interdisciplinarily:

- Mechanical aortic valve was ruled out as unlikely, as this would not have caused hemolysis of the current extent.
- Thrombotic microangiopathy (e.g., hemolytic–uremic syndrome) was ruled out as unlikely since the patient’s platelet count remained stable and was within the normal range (>150,000 cells/µL).
- Disseminated intravascular coagulation (DIC) was thought of as a possible diagnosis, but the leading symptom was extensive hemolysis; there was no sign of thrombosis, and—as mentioned above—platelet count was stable.
- Contamination of the contrast medium was a possible diagnosis, but broad-spectrum antibiotic therapy was already performed.
- Contrast medium-induced hemolysis remained the most likely diagnosis after the above-mentioned considerations.

**Table 2** shows a timeline of the events.

**Table 2:** Timeline for ICU admission, appearance, and treatment of hemolysis and transfer to rehabilitation facility.

Timepoint	Event
POD 0	Surgery 1: mechanical aortic valve implant (ICU admission as planned)
POD 0	Surgery 2: revisional thoracotomy due to hemothorax; implantation of VA-ECMO (later VAV-ECMO)
POD 8	End of ECMO therapy
POD 8–20	Prolonged ICU therapy due to various organ dysfunction
POD 20	CT scan due to symptoms of acute infection; appearance of hemolysis
POD 20	Immediate treatment with prednisolone and eculizumab
POD 20–30	Prolonged ICU therapy due to various organ dysfunction
POD 50	Transfer to rehabilitation facility

POD, post-op day; ICU, intensive care unit; VA-ECMO, veno-arterial extracorporeal membrane oxygenation; VAV-ECMO, veno-arterial venous extracorporeal membrane oxygenation.

## 3 Diagnostic assessment, therapeutic intervention, and outcome

Detailed diagnostic was performed, and the direct anti-globulin test (direct Coombs test) revealed IgG antibodies, C3c, and C3d (complement factors) as evidence for hemolysis (**Supplementary Figure S1B** **).** To test for CM-induced hemolysis, a blood sample was sent to the Center for Transfusion Medicine and Cell Therapy Charité Berlin; test results can be seen in **Box 1**. Further diagnostics and their results can be seen in **Supplementary Table S2**.

Box 1 **Results of the drug-dependent reactions. In the presence of iomeprol, antibodies interact with the red blood cells.**

IAT (standard) negative

**IAT (in the presence of Iomeprol) strongly positive (3+)**

**DAT positive**

 **DAT polyspecific 3+**

 **DAT anti-IgG 3+**

 DAT anti-IgA neg

 DAT anti-IgM neg

 **DAT anti-C3d 3+**

As we were unable to wait for the diagnostic results, CM-induced hemolysis was identified as the most probable diagnosis. A calculated therapy with corticosteroids (1 g methylprednisolone/day for 3 days), intravenous immunoglobulin (IVIG) (80 g, single dose) and eculizumab (900 mg, single dose) were given. Our treatment accomplished quick cessation of hemolysis, and transfusion of red blood cell concentrates could be stopped (cumulatively, 3 L was transfused within 10 hours); lactate cleared within 30 hours. In parallel, the hemodynamic situation and dosage of vasoactive medication remained unchanged.

Immunohematologic in-house examination showed the following results:

- Direct antiglobulin test (DAT) reactive with anti-IgG, anti-C3c, and anti-C3d,
- DAT non-reactive with anti-IgM or anti-IgA,
- eluate (LUI-EICHER) weakly positive with red blood cells A_1_, and
- indirect antiglobulin test (IAT) negative (no auto- or allo-antibodies).

Assessment: The red blood cells of our patient were covered with complement-activating IgG antibodies. In the eluate, an anti-A_1_ antibody was specified, and IAT was negative. The anti-A_1_ antibody represents an irregular cold antibody, existing in approximately 1% of the patients with blood type A_2_. As it prevails at 37°C, the antibody was classified as relevant in further transfusions. The antibody-covered red blood cells were transfused with A_1_ positive red blood cells of the donor. In further transfusions, the patient received only red blood cells of type 0 or A_2_.

A reference immunohematology laboratory specializing in drug-dependent, platelet-reactive antibodies (DDAB) detection confirmed the iomeprol antibody.

**Supplementary Table S3** shows the laboratory findings after the resolution of autoimmune hemolysis on POD 23 (1 day after the onset of hemolysis and administration of eculizumab), POD 26 (3 days after onset of hemolysis and administration of eculizumab), and POD 31 (after the onset of hemolysis and administration of eculizumab). Notably, hemolysis parameters—lactate dehydrogenase (LDH) and free hemoglobin—decreased steadily.

Recovery of our patient was prolonged due to previous and further complications unrelated to CM-induced hemolysis. Seven weeks after his first surgery and 4 weeks after his massive hemolysis, he was able to be transferred to a rehabilitation facility; after 1 year, he was fully fit to work with no restrictions concerning physical or psychological capacity.

## 4 Discussion

### 4.1 Drug-induced hemolysis

The first report about drug-induced immune hemolytic anemia (DIIHA) was published in 1966; however, the exact immunologic mechanism is not fully clarified (5).

The magnitude can vary extremely: in 90% of the cases, acute and intravascular hemolysis should be expected (2). The incidence is approximately 1:1 million (1), mortality is up to 30% (even higher in children) (6), and severe complications appear in approximately 15% (2).

More than 130 drugs have been associated with DIIHA (1); the most frequent drugs involved are antibiotics (2), especially cefotetan and ceftriaxone (5).

Therapy principles are ajar to the treatment of autoimmune hemolytic anemias (7, 8), although the benefits of therapy are dependent on the relationship of treatment to the etiology of the disease (9). The primary therapy of drug-induced hemolysis is composed of the cessation of the culprit drug, blood transfusion, and symptomatic support of the hemodynamic situation. Fewer data exist for steroids, IVIG, plasmapheresis, or immunosuppressants (1, 6).

Different steroid regimens in the treatment of warm autoimmune hemolytic anemia were comparable concerning their safety profile, so parental regimes are used as rescue treatment (10). In drug-induced forms of hemolytic anemia, steroids are given frequently, even without proven benefit.

For cases of drug-independent antibodies, autoantibodies are responsible for hemolysis, and the term cross-reactive autoantibody mechanism has been coined (5). Steroid therapy can be helpful, and IVIG can be tried if there are signs of intravascular hemolysis. These cases are serologically indistinguishable from warm autoimmune hemolytic anemia and treated likewise (6, 11).

Plasmapheresis/plasma exchange has been applied in cases of DIIHA to remove drug-induced antibodies, or in renal failure, where the causative agent could not be eliminated (6).

### 4.2 Mechanisms of DIIHA according to Branch et al.

Branch et al. developed a model that unifies some of the previous concepts and explains different serological findings and the reason why different therapies are necessary.

- Hapten-specific mechanism: hapten (drug) forms a complex via covalent binding (β-lactam group) with a red blood cell (carrier); this complex initiates the production of antibodies against the hapten (**Supplementary Figure S2**, number 1), and plasmapheresis/plasma exchange could be helpful.
- Neoantigen-dependent mechanism: drug and surface of the red blood cell form a neoantigen [drug plus red blood count (RBC) components]; antibody binding is not of very high affinity, and the result is a DAT, which is only positive for C3, but eluates can sometimes be reactive (see **Supplementary Figure S2**, number 2); here, eculizumab could be of value (6, 12).
- Cross-reactive autoantibody mechanism: hapten and red blood cell membrane cause a modified RBC protein, which initiates the production of antibodies reacting with the altered RBC antigen, but also against “normal” RBC. Even without the drug, the production of the antibody and the linking to RBC continues [resemblance with autoantibodies in warm autoimmune hemolytic anemia (wAIHA)], but without the drug, there is no hemolysis (**Supplementary Figure S2**, number 3).
- Immunoglobulin adsorption mechanism: the drug causes various proteins to be bound to the RBC (e.g., IgG), which interact with the mononuclear phagocyte system, causing hemolytic anemia. Again, drugs with a β-lactam group are involved, but this time, other reactive groups mediate the binding to the RBC, and the β-lactam group binds proteins non-specifically.

A synopsis of the different nominations, serological findings, therapy, clinical symptoms, and culprit drugs are shown in **Table 3**.

**Table 3:** Overview of DIIHA (5, 6, 11).

“New” name of mechanism of DIIHA (according to Branch et al. (5)	Hapten-specific mechanism	Neoantigen-dependent mechanism	Cross-reactive autoantibody mechanism	Immunoglobulin adsorption mechanism
Previous name of mechanism of DIIHA	• Drug adsorption “Penicillin type”	• Immune complex mechanism “immune complex type”		• Non-specific protein adsorption Non-immunological protein adsorption (NIPA)
Most common serological findings	• DAT positive for IgG • IAT positive/negative • Eluate negative	• DAT positive for C3 (possible positive for IgM or IgG) • IAT positive/negative • Eluate negative	• DAT positive • IAT positive • Eluate positive • Resembles autoantibodies in wAIHA (13)	• No drug-induced antibodies • DAT positive (unspecific bound IgG interacts with mononuclear phagocyte system) • IAT negative • Eluate negative
Drug-dependent	Yes	Yes	No	No
Therapy	• Stop the drug • No evidence of steroids • Consider plasmapheresis/plasma exchange	• Stop the drug • No evidence of steroids • Case report for IVIG (6) • Consider eculizumab	• Stop the drug • Steroids • IVIG if intravascular hemolysis Immunosuppression	Stop the drug
Probable culprit drugs	High-dose penicillin, Cefotetan (β-lactam group)	Ceftriaxone, piperacillin, NSAIDs	Methyldopa, levodopa, procainamide, fludarabine	Cefotetan, cephalothin, tazobactam (β-lactam group), platinum-based chemotherapeutics
Clinical symptoms/appearance	Extravascular immune hemolysis via RES–macrophage FC–receptor recognition	Intravascular hemolysis, more severe in children	Only hematologic improvement after stopping the culprit drug can confirm DDAB-mediated DIIHA over wAIHA	Extravascular hemolysis in spleen

DAT, direct antiglobulin test; DIIHA, drug-induced immune hemolytic anemia; IAT, indirect antiglobulin test; IVIG, intravenous immunoglobulin; NIPA, non-immunological protein adsorption; NSAIDs, non-steroidal anti-inflammatory drugs; RES, reticuloendothelial system; wAIHA, warm autoimmune hemolytic anemia.

### 4.3 CM-induced hemolysis

In the literature, four case reports of CM-induced hemolysis have been found:

- A female patient with complement-mediated hemolysis after Isopaque and evidence of CM-dependent IgM antibodies (4).
- In a 34-year-old patient with hemolysis following the fourth dose of iomeprol, the decrease in hemoglobin could be treated well with the transfusion of 4 units of red blood cell concentrations without any further complications, and the drug-dependent antibody was of the IgM class (3).
- A 32-year-old patient with metastasized ovarian cancer, hemolysis following the third dose of iomeprol, transfusion of multiple red blood cell units, and short catecholamine support (2).
- A 70-year-old patient with pneumonia had a fatal outcome after a CT scan with iohexol (Omnipaque). However, a septic shock was diagnosed as well (1).

In our case, we were confronted with a massive life-threatening intravascular hemolysis. To our knowledge, no drug-induced hemolysis with this magnitude and successful therapy has been published.

Without knowing the serological findings, we chose a double-barreled therapy with eculizumab for the neoantigen-dependent mechanism and IVIGs plus steroids for the cross-reactive autoantibody mechanism. As the most probable mechanism, the neoantigen-dependent mechanism can be considered in our case (serological findings, clinical appearance, and fast recovery after eculizumab); retrospectively, IVIGs and methylprednisolone would probably have been unnecessary.

However, the actual availability of data is limited, and many details regarding reason, mechanism, and therapy are yet unknown. The recommendation of a single specific therapy according to the clinical appearance or the serological finding is yet not possible.

## 5 Conclusion

A drug-induced hemolytic anemia can show different clinical characteristics. There are different mechanisms and serological findings with specific therapeutic approaches. However, data regarding drug-induced hemolytic anemia are incomplete. We illustrate the diagnosis and therapy of a fulminant intravascular iomeprol-induced hemolytic anemia.

## Data Availability

The original contributions presented in the study are included in the article/**Supplementary Material**. Further inquiries can be directed to the corresponding author.

## Supplementary material

Supplementary Figure 1 Clinical presentation and laboratory findings of the immune hemolysis: **(A)** Blood samples before (from left to right: tube 1-3) and after the hemolysis (from left to right: tube 4-6). **(B)** IgG-antibodies, C3c and C3d (complement factors) as evidence for hemolysis.

Supplementary Figure 2 Schematic mechanism of a red blood cell as a carrier for a drug operation as a hapten (according to (5)): No. 1 pictures the hapten-dependent mechanism, where the hapten (drug) forms a complex via covalent binding (ß-lactam group) with a red blood cell (carrier); this complex initiates the production of antibodies against the hapten. No. 2 presents the neoantigen-dependent mechanism, where drug and surface of the red blood cell form a neoantigen (drug plus RBC components) (number 2). No. 3 shows the cross-reactive autoantibody mechanism, where hapten and red blood cell membrane cause a modified RBC protein, which initiates production of antibodies reacting with the altered RBC antigen, but also against “normal” RBC. Even without the drug, the production of the antibody and the linking to RBS continues (resemblance with autoantibodies in wAIHA), but without the drug, there is no hemolysis.
