# Supplementary figures and images for: A hyper-acute immune hemolytic anemia induced by contrast medium was successfully treated with eculizumab: a case report
Source: Front Immunol. 2025 Feb 11;16:1464014. doi: 10.3389/fimmu.2025.1464014 (PMC11850351; doi:10.3389/fimmu.2025.1464014)

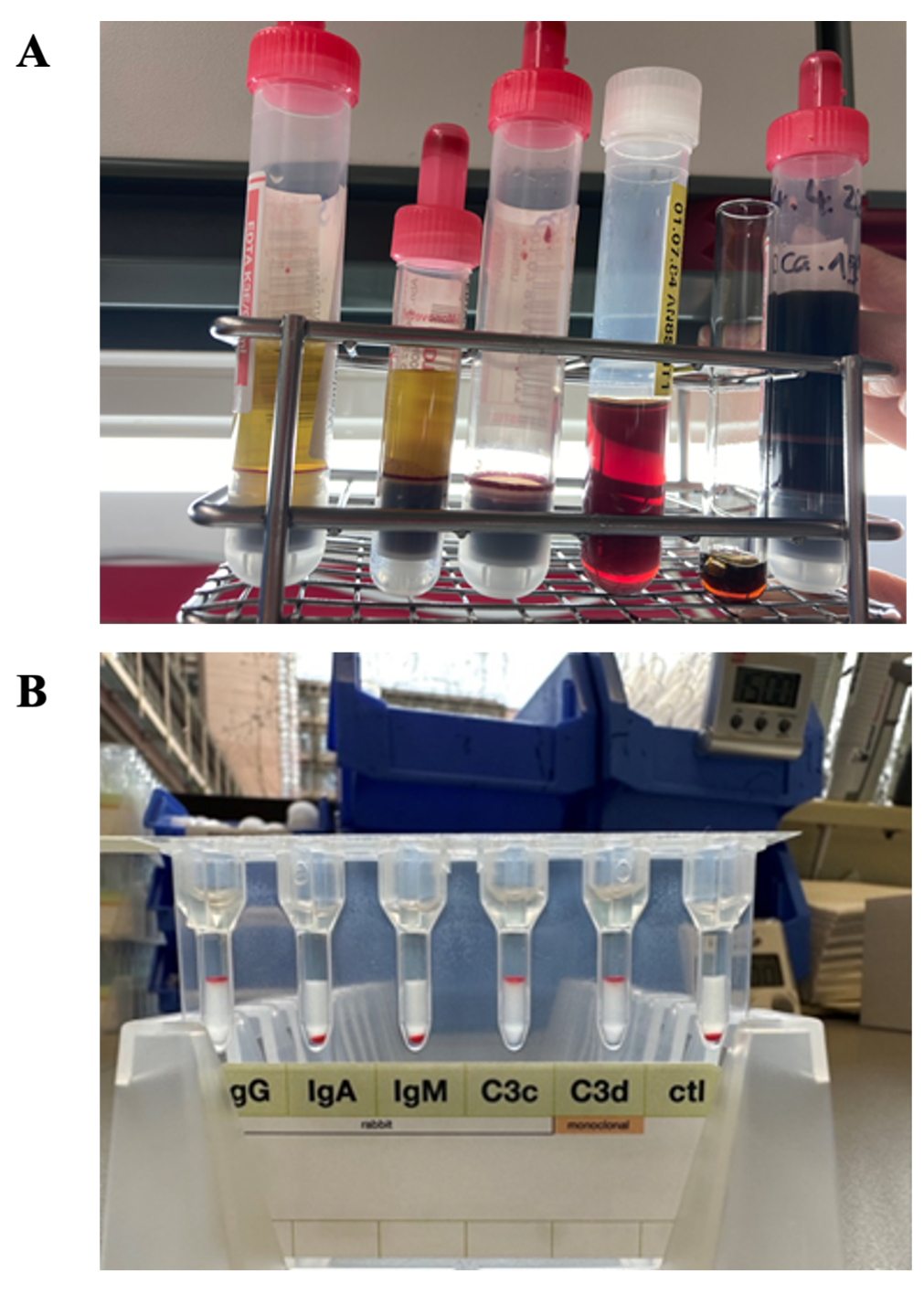

Supplement: Supplementary Figure 1 — Clinical presentation and laboratory findings of the immune hemolysis: (A) Blood samples before (from left to right: tube 1-3) and after the hemolysis (from left to right: tube 4-6). (B) IgG-antibodies, C3c and C3d (complement factors) as evidence for hemolysis. [file Image1.tiff]

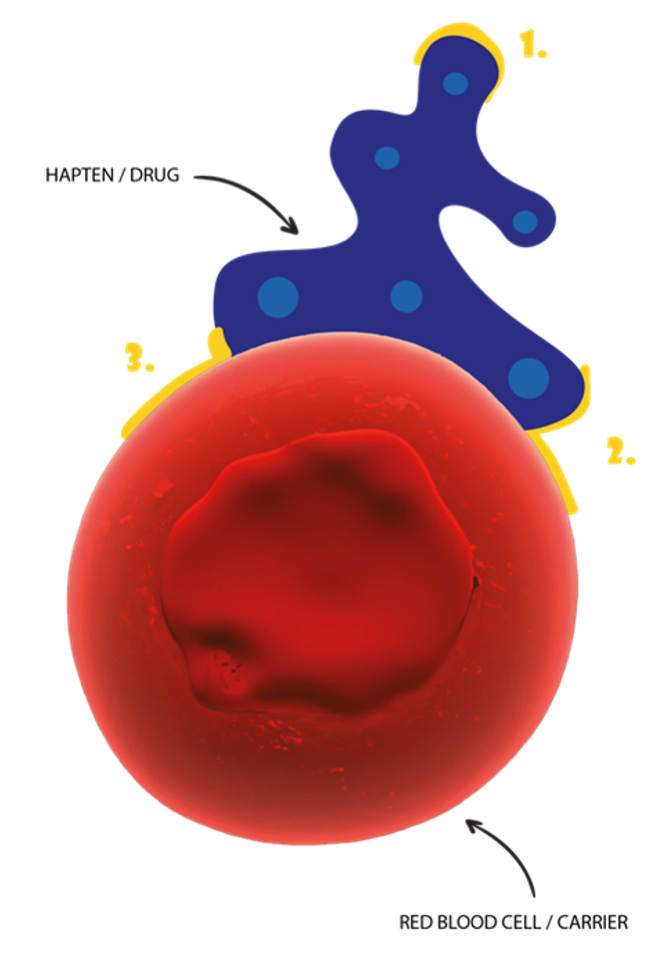

Supplement: Supplementary Figure 2 — Schematic mechanism of a red blood cell as a carrier for a drug operation as a hapten (according to (5)): No. 1 pictures the hapten-dependent mechanism, where the hapten (drug) forms a complex via covalent binding (ß-lactam group) with a red blood cell (carrier); this complex initiates the production of antibodies against the hapten. No. 2 presents the neoantigen-dependent mechanism, where drug and surface of the red blood cell form a neoantigen (drug plus RBC components) (number 2). No. 3 shows the cross-reactive autoantibody mechanism, where hapten and red blood cell membrane cause a modified RBC protein, which initiates production of antibodies reacting with the altered RBC antigen, but also against “normal” RBC. Even without the drug, the production of the antibody and the linking to RBS continues (resemblance with autoantibodies in wAIHA), but without the drug, there is no hemolysis. [file Image2.tiff]
