# Supplementary material for: A hyper-acute immune hemolytic anemia induced by contrast medium was successfully treated with eculizumab: a case report
Source: Front Immunol. 2025 Feb 11;16:1464014. doi: 10.3389/fimmu.2025.1464014 (PMC11850351; doi:10.3389/fimmu.2025.1464014)
Supplement: Supplementary file 3 [file Table1.docx]

| **Year** | **Surgery/intervention** |
| --- | --- |
| 1984 (newborn) | Resection of the preductal aortic isthmus stenosis with end-to-end anastomosis |
| 1999 (age 15) | Balloon angioplasty |
| 2000 (age 16) | Ballon angioplasty |
| 2000 (age 16) | Replacement of the proximal descending aorta with implantation of the left subclavian artery |
| 2008 (age 24) | Reconstruction of the aortic valve |

**Table 1S:** Patient’s detailed surgical history
