# Supplementary material for: A hyper-acute immune hemolytic anemia induced by contrast medium was successfully treated with eculizumab: a case report
Source: Front Immunol. 2025 Feb 11;16:1464014. doi: 10.3389/fimmu.2025.1464014 (PMC11850351; doi:10.3389/fimmu.2025.1464014)
Supplement: Supplementary file 4 [file Table2.docx]

| **Test** | **Results** | **Interpretation** |
| --- | --- | --- |
| Indirect anti-globulin test (indirect Coombs-test) | no evidence of irregular antibodies up to two month after hemolysis |  |
| Cell morphology | schistocytes 9‰ | difficult quantification of schistocytes, because of strong agglutination of red blood cells), no reliable result for/against TMA |
| ADAMTS 13 | normal | TTP unlikely |
| HIT II | negative |  |
| Microbiological testing of CM bottle | no contamination found |  |

**Table 2S:** Further results of our diagnostic measures.

CM: contrast medium; HIT II: heparin-induced thrombocytopenia Type II; TMA: thrombotic microangiopathy; TTP: thrombotic-thrombocytopenic purpura
