# Supplementary material for: A hyper-acute immune hemolytic anemia induced by contrast medium was successfully treated with eculizumab: a case report
Source: Front Immunol. 2025 Feb 11;16:1464014. doi: 10.3389/fimmu.2025.1464014 (PMC11850351; doi:10.3389/fimmu.2025.1464014)
Supplement: Supplementary file 5 [file Table3.docx]

| **Laboratory findings** | **Normal range** | **POD 23** | **POD 26** | **POD 31** |
| --- | --- | --- | --- | --- |
| Hemoglobin g/dl | 12.0-16.0 | 13.9 | 8.9 | 7.9 |
| Hematocrit % | 42.0 – 52.0 | 40.0 | 24.3 | 23.1 |
| Erythrocytes Mio/µl | 4.2-6.2 | 4.62 | 2.89 | 2.63 |
| Platelet count 1000/µl | 150-450 | 151 | 54 | 79 |
| leukocyte 1/µl | 4100-11800 | 29009 | 24360 | 11690 |
| aPTT sec | <40 | 94 | 69 | 57 |
| INR |  | 2.0 | 1.1 | 1.1 |
| D-dimer µl/ml FE | 0-0.5 | >80 | 58 |  |
| Creatinine mg/dl | 0.6-1.1 | 1.6 | 0.8 | 1.4 |
| Total bilirubin mg/dl | <1.1 | 6.5 | 18.0 | 7.0 |
| CRP mg/dl | <0.5 | 9.68 |  | 3.79 |
| PCT ng/ml | <0.1 | >50.00 |  | 4.63 |
| IL-6 ng/l | 0-4 | 86.6 |  | 32.6 |
| CK U/l | <170 | hemolytic | 771 | 125 |
| LDH U/l | <250 | hemolytic | 8498 | 857 |
| Free hemoglobin mg/dl | <10 | 432.52 | 90.52 | 15.34 |
| Haptoglobin mg/dl | <6 | <6 | <6 |  |

**Table 3S:** Laboratory findings after resolution of autoimmune hemloysis on POD 23 (one day after onset of hemolysis and administration of Eculizumab), POD 26 (three days after onset of hemolysis and administration of Eculizumab), and POD 31 (after onset of hemolysis and administration of Eculizumab).
